# Neutral theory: applicability and neutrality of clinical study endpoints where a disease-specific instrument is available

**DOI:** 10.1186/s12874-023-01947-z

**Published:** 2023-05-20

**Authors:** Ravi Jandhyala

**Affiliations:** 1 Medialis Ltd, 3 Warren Yard, Wolverton Mill, Milton Keynes, MK12 5NW UK; 2 Centre for Pharmaceutical Medicine Research, King’s College University, London, UK

**Keywords:** Neutral theory, Clinical trial, Rare disease, Disease-severity measurement, Accuracy, Patient misclassification, Trial recruitment

## Abstract

**Background:**

There is a pressing need to improve the accuracy of rare disease clinical study endpoints. Neutral theory, first described here, can be used to assess the accuracy of endpoints and improve their selection in rare disease clinical studies, reducing the risk of patient misclassification.

**Methods:**

Neutral theory was used to assess the accuracy of rare disease clinical study endpoints and the resulting probability of false positive and false negative classifications at different disease prevalence rates. Search strings were extracted from the Orphanet Register of Rare Diseases using a proprietary algorithm to conduct a systematic review of studies published until January 2021. Overall, 11 rare diseases with one disease-specific disease severity scale (133 studies) and 12 rare diseases with more than one disease-specific disease severity scale (483 studies) were included. All indicators from clinical studies were extracted, and Neutral theory was used to calculate their match to disease-specific disease severity scales, which were used as surrogates for the disease phenotype. For those with more than one disease-severity scale, endpoints were compared with the first disease-specific disease severity scale and a composite of all later scales. A Neutrality score of > 1.50 was considered acceptable.

**Results:**

Around half the clinical studies for half the rare diseases with one disease-specific disease severity score (palmoplantar psoriasis, achalasia, systemic lupus erythematosus, systemic sclerosis and Fournier’s gangrene) met the threshold for an acceptable match to the disease phenotype, one rare disease (Guillain-Barré syndrome) had one study with an acceptable match, and four diseases (Behcet’s syndrome, Creutzfeldt-Jakob disease, atypical hemolytic uremic syndrome and Prader-Willi syndrome) had no studies. Clinical study endpoints in almost half the rare diseases with more than one disease-specific DSS (acromegaly, amyotrophic lateral sclerosis, cystic fibrosis, Fabry disease and juvenile rheumatoid arthritis) were a better match to the composite, while endpoints in the remaining rare diseases (Charcot Marie Tooth disease, Gaucher disease Type I, Huntington’s disease, Sjogren’s syndrome and Tourette syndrome) were a worse match. Misclassifications varied with increasing disease prevalence.

**Conclusions:**

Neutral theory confirmed that disease-severity measurement needs improvement in rare disease clinical studies, especially for some diseases, and suggested that the potential for accuracy increases as the body of knowledge on a disease increases. Using Neutral theory to benchmark disease-severity measurement in rare disease clinical studies may reduce the risk of misclassification, ensuring that recruitment and treatment effect assessment optimise medicine adoption and benefit patients.

**Supplementary Information:**

The online version contains supplementary material available at 10.1186/s12874-023-01947-z.

## Background

In the European Union and the United Kingdom, rare diseases are defined as those affecting 1 in 2000 people and in the United States, fewer than 200,000 [1–3]. Although they collectively affect up to 1 in 17 people in the UK alone, as many as 90% of rare diseases have no established treatment [4]. Conducting robust rare disease clinical trials is challenging due to disease heterogeneity and geographic dispersion, low power by default, and a poor understanding of disease progression despite growing research attention [5–7]. As a result, clinical trials in this population may deliver weak safety and efficacy evidence, which along with the high cost of orphan medicines, may lead to patient access issues [8]. Additionally, almost 10% (33 of 353) of orphan drugs that received marketing authorisation between 1983 and 2010 were not marketed or were withdrawn, and almost half had no equivalent in the target therapeutic indication [8]. With the high levels of unmet need and economic and psychosocial burden in the rare disease community, the cost to patients of clinical trial failure can be immense [4]. These circumstances suggest a need to optimise rare disease clinical trial practice to ensure that every trial has the best chance of delivering results that lead to medicine adoption and patient benefit, which can be achieved through accurate construct measurement and the selection of endpoints relevant to patients and gatekeepers [9, 10].

For a medicine to reach patients, regulators and payors must be convinced by clinical trial and real-world evidence submitted for marketing authorisation and pricing and reimbursement, and prescribers must be convinced by evidence sufficient to inform treatment decisions [9]. Such evidence may be generated using generic measures of disease severity, for example, as entry criteria and to stratify patients for clinical trials [11] and to determine treatment effects. Yet, most generic measurement tools for disease severity are inadequate in addressing comorbidity [12], high levels of which are common in rare diseases. For example, generic pain measures are inadequate in rare neuromuscular and musculoskeletal disease; multidimensional/rare-disease specific measures are needed to measure pain phenotype accurately [13]. Generic DSSs did not accurately predict mortality in COVID-19 and are therefore unsuitable for stratification in clinical trials [11]. The lack of concordance between generic and specific quality of life measures means that generic scales may be insufficiently specific and sensitive to detect treatment effects in clinical trials, even when they are present [14, 15]. The inadequacy of generic disease-severity scales in rare diseases means that similar issues are expected with their use in clinical studies. Additionally, variation in the accuracy of disease-severity measurement may influence trial recruitment. For example, one study showed that as many as 20% of patients could have been excluded due to inter-trial variation in disease-severity measurement [16].

There are currently no standardised methods for assessing the accuracy of disease construct measurement in clinical studies. Neutral theory, first described here, is a possible solution rooted in the philosophy of measurement. Fallibilism, especially that proposed by Peirce [17], suggests that all measurable scientific constructs are merely probabilistic theoretical approximations of an underlying and absolute ‘true’ object. Additionally, Peirce argued that to have a true, pragmatic understanding of a concept, it is necessary to understand the effects expected if held true [17]. In the context of disease measurement, the absolute ‘true’ object can be considered the disease phenotype, which can be expressed as all observable indicators of a disease. In this case, a disease indicator can be understood as a manifest characteristic of an underlying disease condition. Neutral theory suggests that all measurements of disease constructs, such as disease severity, are accurate only to the extent that the indicators included match those in the disease phenotype (the Neutral list of indicators). In other words, a truly unbiased (‘neutral’) measure of disease constructs can be achieved by measuring all indicators in the disease phenotype and avoiding those that are excluded [18].

The aim of this study was to apply Neutral theory to assess the accuracy of disease-severity measurement in rare disease clinical studies and the expected rates of false positive and false negative classifications if held true.

## Methods

### Systematic review to identify clinical studies and rare diseases with disease-specific disease-severity scales

A systematic review was conducted to identify rare disease clinical studies and rare diseases with disease-specific DSSs, which were used as surrogates for the disease phenotype. The review was conducted according to the preferred reporting items for systematic reviews and meta-analysis (PRISMA) guidelines on the Medline (PubMed) database, including all studies published until 21 January 2021. For each disease, the review used generic search strings as well as specific search strings generated from the Orphanet Register of Rare Diseases (https://www.orpha.net/consor/cgi-bin/Disease_Search_List.php?lng=EN) using a proprietary algorithm. All search strings have been provided in Supplemental Table 1. To be included, studies had to be randomised controlled trials or observational clinical studies, conducted with human participants, have clearly recorded outcomes/endpoints, have full text available and be written in the English language. For a disease to be included in the study, there had to be at least one associated disease-specific DSS and more than five studies that used each of its associated DSSs to measure the severity of the disease in published literature. The studies also had to state clearly the disease studied. Two research analysts conducted the reviews, and the initial screening of titles and abstracts was conducted using Rayyan [19], a web-based tool that automates systematic review processes.

**Table 1:** Clinical study indicators in the calculation of Neutrality

Indicators in a clinical study	Indicators within DSS	Indicators outside DSS
Included	Overlap (a)	Redundant (b)
Excluded	Missing (c)	Irrelevant (d)
Total	(a + c)	(b + d)
**Neutrality (accuracy)**	*Sensitivity: a/a* + *c*	*Specificity: d/b* + *d*

### Rare diseases with one disease-specific severity scale and those with more than one

Rare diseases were categorised into two groups, those with one disease-specific DSS and those with more than one. For the first group, the match between clinical study endpoints for each rare disease and its disease-specific DSS was calculated. For the second group, the match between clinical study endpoints and the first disease-specific DSS was compared with the match between clinical study endpoints and a composite of all later disease-specific DSSs. To create the composite, we extracted all indicators from later disease-specific DSSs and excluded duplicates. Indicators from the composite were included as part of the total information observed in the analysis for the first disease-specific DSS (Table 1).

### Using Neutral theory to determine the accuracy of clinical study disease measurement

**Fig. 1:**
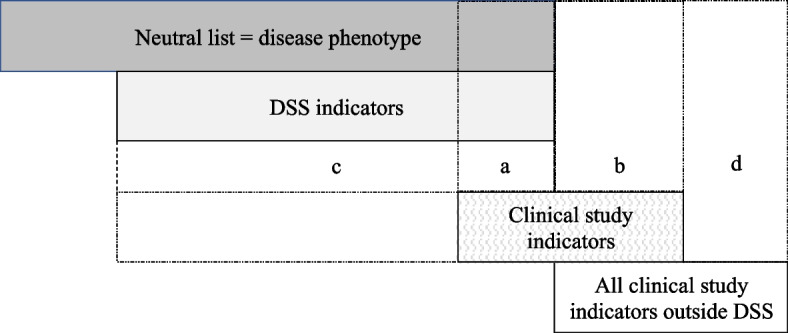
Selection of articles for use to assess the neutrality of clinical studies

Neutral theory was applied to determine the accuracy of clinical study disease measurement, which was assessed as the extent to which clinical study endpoints matched disease-specific DSSs (Fig. 1). The extent to which rare disease clinical study endpoints matched the disease phenotype was determined by calculating the match between indicators extracted from clinical studies and disease-specific DSSs, which were used as surrogates for the disease phenotype. Indicators included in both the study and DSS were classified as *overlapping*, those present in only the study but not the score were classified as *redundant*, and those present in only the score but not the study were classified as *missing*. Indicators not in the study or DSS, but included as an endpoint in another study of the same disease were classified as *irrelevant*. These classifications were used to calculate the Neutrality of clinical study endpoints, which was defined in terms of sensitivity and specificity (Fig. 1, Table 1).

Sensitivity was defined as the proportion of *overlapping* indicators among the total disease-specific score items (a/a + c), and specificity was defined as the proportion of *irrelevant* indicators among the total items outside disease-specific score items (d/b + d). A Neutrality score was calculated, which was the sum of the sensitivity and specificity, with a score of 2 representing perfect Neutrality (100% sensitivity and 100% specificity) and > 1.50 good Neutrality. Positive and negative predictive values are calculated using the Neutrality of a choice of items (sensitivity and specificity) and the prevalence of severe disease (set at 20%, 50%, and 80%). The rates of false negatives and false positives (calculated based on negative and positive predictive values, respectively) were evaluated using the sample sizes as reported in the clinical studies. Sensitivity and specificity were treated as statistically independent. All analyses were performed using R 3.6.0 (R Core Team, 2021) and Microsoft Excel 365 (Microsoft).

## Results

### Rare diseases and clinical studies with one disease-specific disease severity scale

**Fig. 2:**
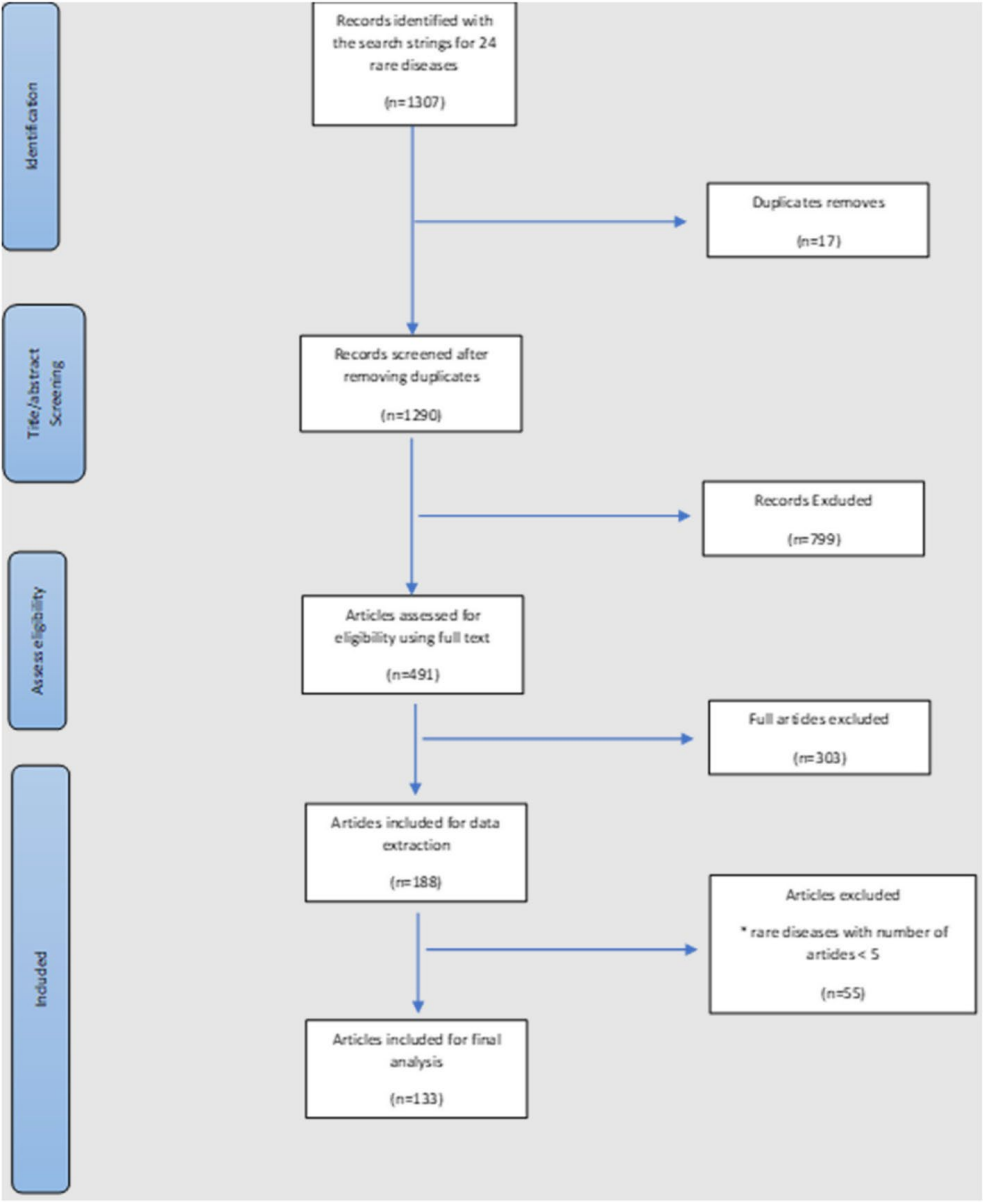
Potential misclassifications for all diseases in respect of the first DSS

Overall, 133 of the 1290 studies reviewed were included (Fig. 2). A total of 24 rare diseases with a single, validated disease-specific DSS were identified. Of these, 11 had at least five studies assessing disease severity. Hyperhidrosis was excluded, as the disease can be primary (idiopathic) or secondary to another condition, and there was a lack of clarification on what aetiology was being assessed for severity. Thus, 10 rare diseases with one disease-specific DSS were included: achalasia, Behcet’s syndrome, Creutzfeldt-Jakob disease, Fournier’s gangrene, Guillain-Barré syndrome, atypical hemolytic uremic syndrome, palmoplantar psoriasis, Prader-Willi syndrome, systemic lupus erythematosus, systemic sclerosis (Table 2).

**Table 2:** Overview of rare disease with one disease-specific disease severity scale

Disease	Disease-specific DSS	Number of indicators
Achalasia	Eckardt Score	40
Behcet’s syndrome	Krause's Behçet's Disease Activity Assessment	38
Creutzfeldt-Jakob disease	Creutzfeldt-Jakob Disease Neurological Status Scale	29
Fournier’s gangrene	Fournier’s Gangrene Severity Index	20
Guillain-Barré syndrome	Guillain-Barré Rating Scale	20
Atypical hemolytic uremic syndrome	Pediatric Neurologic Assessment Score for Hemolytic Uremic Syndrome	28
Palmoplantar psoriasis	Palmoplantar Psoriasis Area and Severity Index	3
Prader-Willi syndrome	Prader-Willi Syndrome Behavioral Questionnaire	39
Systemic lupus erythematosus	Lupus Severity Index	48
Systemic sclerosis	Modified Rodnan Skin Score	38

### Rare diseases and clinical studies with more than one disease-specific disease severity scale

**Fig. 3:**
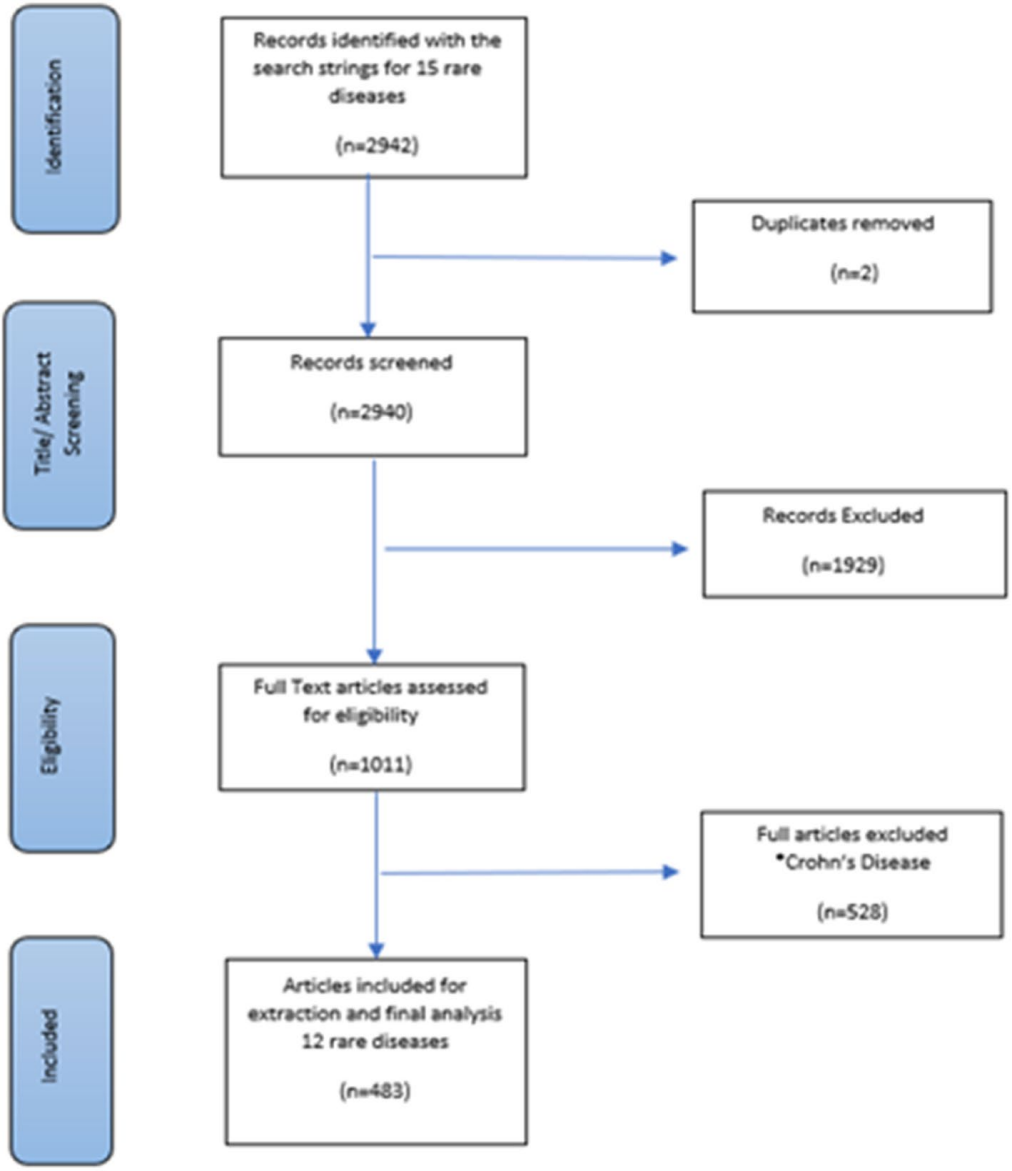
Potential misclassifications for all diseases in respect of the composite DSS

Overall, 483 of the 2942 studies reviewed were included (Fig. 3). A total of 15 rare diseases were identified as having more than one disease-specific DSS. Gaucher disease Type 3 and Niemann Pick disease were identified in early screening as having more than one disease-specific DSS but were eventually excluded due to insufficient published data on their disease-severity measures. Crohn’s disease had more than one disease-specific DSS, but research using them did not distinguish between the disease measured, describing patients as having inflammatory bowel disease or a combination of both Crohn’s and ulcerative colitis. Ulcerative colitis was excluded before review, as there was insufficient published data using its disease-specific DSSs. The following 12 rare diseases were included (number of disease-specific DSSs): acromegaly (3), amyotrophic lateral sclerosis (6), Charcot Marie Tooth disease (5), cystic fibrosis (5), encephalitis (4), Fabry disease (2), Friedreich ataxia (3), Gaucher disease Type 1 (2), Huntington’s disease (2), juvenile rheumatoid arthritis (2), Sjogren’s syndrome (2), and Tourette syndrome (3). An overview of the diseases, the composition and publication dates of their first and composite DSSs and the number of indicators in each has been provided in Table 2. Full details of indicators included in first and composite disease-specific DSSs are available upon reasonable request. As shown in Table 3, the number of unique indicators available as the surrogate disease phenotype for all diseases increased over time.

**Table 3:** Overview of rare diseases and first and composite disease-specific disease severity scales (year of publication)

Disease	First disease-specific DSS	Composite disease-specific DSS
	[total number of indicators]	[total number of indicators, number of duplicates excluded]
Acromegaly	Clinical Activity Score of Acromegaly (1992) [17]	Clinical Activity Score of Acromegaly (1992)
		Acroscore (2015)
		ACRODAT® (2017) [3, 26]
Amyotrophic lateral sclerosis	Appel ALS Rating Scale (1987) [20]	Appel ALS Rating Scale (1987)
		Amyotrophic Lateral Sclerosis Severity Score (1989)
		Modified Norris Scale (1996)
		Amyotrophic Lateral Sclerosis Functional Rating Scale (1999)
		Amyotrophic Lateral Sclerosis Utility Index (2005)
		Japan ALS Severity Classification (2012) [71, 7]
Charcot Marie Tooth disease	Charcot-Marie-Tooth Neuropathy Score (2007) [9]	Charcot-Marie-Tooth Neuropathy Score (2007)
		CMT Neuropathy Score (2011)
		CMT Examination Score (2011)
		CMT Pediatric Scale (2012)
		Mobility-Disability Severity Index (2014) [16, 26]
Cystic fibrosis	The Shwachman-Kulczycki Score (1958) [20]	The Shwachman-Kulczycki Score (1958)
		Brasfield Score (1979)
		Cystic Fibrosis Clinical Score (1999)
		Matouk Clinical Score (2004)
		Chrispin–Norman Score (2005) [61, 10]
Encephalitis	The Status Epilepticus Severity Score (2008) [4]	The Status Epilepticus Severity Score (2008)
		END IT Score (2016)
		The Epidemiology-Based Mortality Score in Status Epilepticus (2015)
		The Clinical Assessment Scale in Autoimmune Encephalitis (2019) [43, 2]
Fabry disease	The Mainz Severity Score Index (2003) [25]	The Mainz Severity Score Index (2003)
		FD Severity Scoring System (2009) [37, 0]
Friedreich ataxia	The International Cooperative Ataxia Rating Scale (1997) [24]	The International Cooperative Ataxia Rating Scale (1997)
		The Scale for the Assessment and Rating of Ataxia (2004)
		Friedreich Ataxia Rating Scale (2010) [50, 6]
Gaucher disease type I	Gaucher Disease Severity Score Index – Type I (2008) [15]	Gaucher Disease Severity Score Index – Type I (2008)
		The Disease Severity Scoring System (2015) [1, 25]
Huntington’s disease	The Unified Huntington’s Disease Rating Scale Motor Score (2013) [7]	The Unified Huntington’s Disease Rating Scale Motor Score (2013)
		Problem Behaviors Assessment for Huntington Disease (2015) [18, 0]
Juvenile rheumatoid arthritis	Clinical Disease Activity Index for RA (2005) [4]	Clinical Disease Activity Index for RA (2005)
		Juvenile Arthritis Disease Activity Score (2009) [8, 0]
Sjogren’s syndrome	EULAR Sjögren’s Syndrome Disease Activity Index (2010) [6]	EULAR Sjögren’s Syndrome Disease Activity Index (2010) [6]
		The Sjögren’s International Collaborative Clinical Alliance Ocular Staining Score (2015) [11, 0]
Tourette syndrome	Yale Global Tic Severity Scale (1977) [15]	Yale Global Tic Severity Scale (1977) [15]
		The Shapiro Tourette Syndrome Severity Scale Score (2004)
		Premonitory Urge for Tic Disorders Scale (2012) [28, 0]

### Match between clinical study endpoints and disease phenotype for rare diseases with one disease-specific disease severity score

Half the diseases with one disease-specific DSS (achalasia, Fournier’s gangrene, palmoplantar psoriasis, systemic lupus erythematosus, and systemic sclerosis) had at least one clinical study that was a perfect match for the surrogate disease phenotype with a maximum Neutrality score of 2. Overall match between clinical study endpoints and the disease phenotype was highest for achalasia (Neutrality: 0.86–2.00; sensitivity: 0–100%; specificity: 86–100%), followed by systemic lupus erythematosus (0.83–2.00; 4–100%; 79–100%) and systemic sclerosis (0.82–2.00; 0–100%; 82–100%). Of these diseases, Fournier’s gangrene (Neutrality: 0.45–2.00; sensitivity: 0–100%; specificity: 45–100%) and palmoplantar psoriasis (0.00–2.00; 0–100%; 0–100%) had the worst match to the disease phenotype (Table 4). Supplemental Table 5 shows endpoints used in the five clinical studies included for Fournier’s gangrene showing distribution of endpoints within and outside Fournier’s Gangrene Severity Index (FGSI). Standardisation of validated endpoints was observed in studies using the FGSI, providing a basis for comparative decision-making by stakeholders. However, the need for a Neutral list can be seen in the inclusion of outcomes of relevance to patient and clinician decision-making (such as mortality and quality of life) in other studies.

**Table 4:** Mean neutrality (sensitivity, specificity) of clinical studies measured against disease-specific disease severity scales

Rare disease	Number of papers	Neutrality	
		Most Neutral Study	Least Neutral Study
Achalasia	27	2.00 (1.00, 1.00)	0.86 (0.00, 0.86)
Behcet’s disease	21	1.10 (0.10, 1.00)	0.61 (0.00, 0.61)
Creutzfeldt-Jakob disease	5	0.87 (0.12, 0.75)	0.75 (0.00, 0.75)
Fournier’s gangrene	5	2.00 (1.00, 1.00)	0.45 (0.00, 0.45)
Guillain–Barre syndrome	6	1.80 (0.91, 0.89)	0.33 (0.00, 0.33)
Atypical hemolytic uremic syndrome	6	0.71 (0.08, 0.62)	0.44 (0.00, 0.44)
Palmoplantar pustulosis	5	2.00 (1.00, 1.00)	0.00 (0.00, 0.00)
Prader-Willi syndrome	8	1.02 (0.11, 091)	0.49 (0.04, 0.45)
Systemic lupus erythematosus	22	2.00 (1.00, 1.00)	0.83 (0.04, 0,79)
Systemic sclerosis	28	2.00 (1.00, 1.00)	0.82 (0.00, 0.82)

**Table 5:** Mean neutrality (sensitivity, specificity) of clinical studies measured against first and composite disease-specific disease severity scales

Rare disease	Number of papers	Average neutrality	
		First DSS only	Composite DSS
Acromegaly	30	0.899 (0.029, 0.870)	1.034 (0.110, 0.923)
Amyotrophic lateral sclerosis	81	1.044 (0.109, 0.935)	1.138 (0.166, 0.972)
Charcot Marie tooth	12	1.371 (0.472, 0.898)	1.117 (0.215, 0.902)
Cystic fibrosis	156	0.991 (0.008, 0.983)	1.013 (0.026, 0.987)
Encephalitis	12	0.956 (0.000, 0.956)	0.905 (0.000, 0.905)
Fabry disease	15	0.935 (0.039, 0.896)	1.018 (0.105, 0.913)
Friedreich ataxia	18	1.102 (0.299, 0.803)	1.248 (0.344, 0.904)
Gaucher type I	15	0.969 (0.142, 0.851)	0.943 (0.112, 0.831)
Huntington’s disease	32	1.456 (0.500, 0.956)	1.162 (0.205, 0.957)
Juvenile rheumatoid arthritis	39	0.983 (0.032, 0.951)	1.109 (0.147, 0.962)
Sjogren’s syndrome	37	1.323 (0.401, 0.922)	1.146 (0.224, 0.922)
Tourette’s syndrome	36	1.784 (0.837, 0.946)	1.395 (0.448, 0.946)

Around half the clinical studies for each of these diseases met the threshold for an acceptable match to the disease phenotype (Neutrality score > 1.50). Palmoplantar psoriasis had the highest proportion of such studies (80%; 4/8), followed by achalasia (66.7%; 18/27), systemic lupus erythematosus (59.1%; 13/22), systemic sclerosis (42.9%; 12/28) and Fournier’s gangrene (40.0%; 2/5) (Table 4). Of the remaining diseases/conditions, only one study (16.7%; 1/6) for Guillain-Barré syndrome had an acceptable match to the disease phenotype with a Neutrality score of > 1.50, whilst none of the studies for Behcet’s syndrome (0/21), Creutzfeldt-Jakob disease (0/5), atypical hemolytic uremic syndrome (0/6), and Prader-Willi syndrome (0/8) met this threshold.

### Match between clinical study endpoints and disease phenotype for rare diseases with more than one disease-specific disease severity score

Only one disease (Tourette syndrome) had an acceptable level of Neutrality in at least one study. For almost half the diseases (acromegaly, amyotrophic lateral sclerosis, cystic fibrosis, Fabry disease, and juvenile rheumatoid arthritis), clinical study endpoints were a better match to the composite than the first disease-specific DSS (Table 5). The magnitude of increase in the mean Neutrality of clinical studies showed how much better the match was, with the biggest increase for acromegaly (0.135) and juvenile rheumatoid arthritis (0.126), followed by amyotrophic lateral sclerosis (0.094), Fabry disease (0.083) and cystic fibrosis (0.022). This change appeared to be driven by an increase in the selection of relevant indicators as clinical endpoints, as in most of these diseases, sensitivity increased threefold, but for juvenile rheumatoid arthritis, it was closer to a fivefold increase. Compared to this, there did not seem to be much change in the number of irrelevant indicators excluded, as the increase in the mean specificity of clinical endpoints for each of these diseases was negligible. For Friedreich ataxia, clinical study endpoints were a better match to the composite than the first disease-specific DSS, with the change being driven by sensitivity and specificity to a similar degree.

For the remaining diseases (Charcot Marie Tooth disease, Gaucher disease Type I, Huntington’s disease, Sjogren’s syndrome, and Tourette syndrome), clinical study endpoints were a worse match to composite than the first disease-specific DSS. The magnitude of decrease in the mean Neutrality showed how much worse the match was, with the worst match for Tourette syndrome (-0.389), followed by Huntington’s disease (-0.294), Charcot Marie Tooth disease (-0.254), Sjogren’s syndrome (-0.177) and Gaucher disease Type I (-0.026). As for the other group of diseases, this appeared to be driven by a change in the number of relevant indicators included, as the sensitivity of clinical studies for most of these studies halved. For Gaucher disease Type I, the decrease was much more modest. As before, compared to the decrease in sensitivity, the decrease in specificity was negligible. Clinical endpoints for encephalitis were a slightly worse match to the composite than the first disease-specific DSS, but sensitivity remained constant at zero, while specificity decreased by a modest degree like that shown by the other diseases.

### False positive and false negative classifications arising from mismatch between clinical endpoints and the disease phenotype

**Fig. 4:**
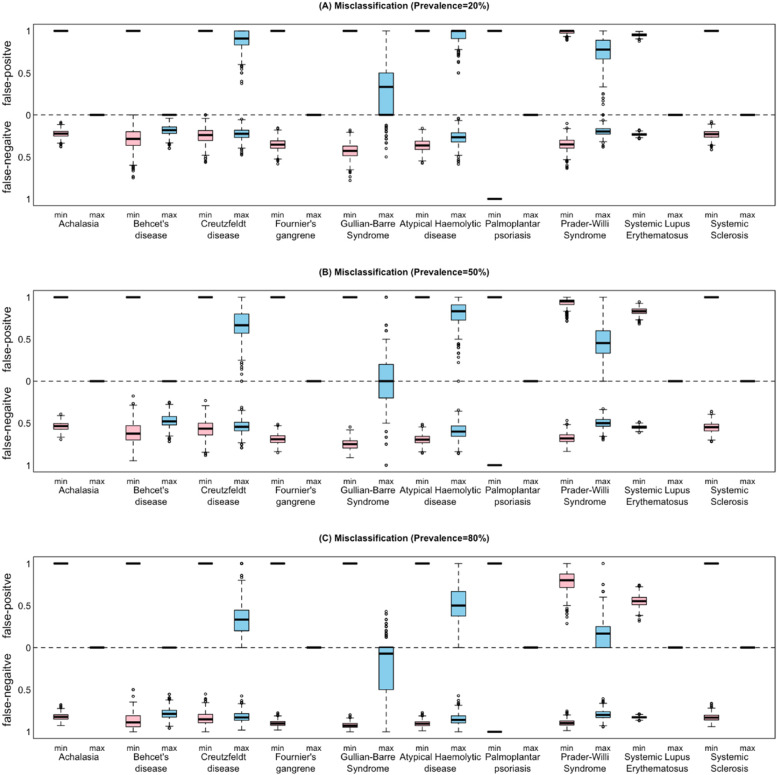
A novel presentation of false positive and false negative rates at 20%, 50% and 80% prevalence of severe disease for the most and least Neutral studies for each disease with one disease-specific disease severity scale. The horizonal dotted line at value 0 represents the target neutrality of 2. For each paper, the number of subjects reported was used to generate the boxplot. The simulation used 1000 replicates. For papers that did not report the number of subjects, a sample size of 30 subjects was used

Few *overlapping* indicators and a high number of *missing* indicators in the study design were reflected as high rates of false positives and negatives (Fig. 4). Conversely, a low level of *missing* indicators reflected a low rate of false negatives, while few *redundant* indicators reflected a low false-positive rate. Overall, the rate of false negatives increased and the rate of false positives decreased with increasing disease-severity prevalence. Supplemental Figs. 1 and 2 show the potential false positive and false negative classifications arising from the neutrality, sensitivity and specificity of clinical studies when assessed against the first and composite DSS. For both first and composite DSSs, the probability of a false negative result increased with increasing disease prevalence, while the probability of a false positive decreased. For both first and composite DSSs, the probability that the least neutral clinical study for most diseases would yield a false positive result was equal to one at all disease prevalence rates. However, for encephalitis, this was true for both the most and least neutral study. There were no instances of the probability of a false negative equalling one for either the first or composite DSS.

## Discussion

There is a pressing need to improve the accuracy of clinical study endpoints in rare diseases [6]. One way to do so is to ensure that clinical study endpoints reflect disease phenotypes. This study examined the extent to which endpoints in clinical studies for rare diseases matched disease-specific DSSs, which were used as surrogates for disease phenotypes. We expected that the match between clinical endpoints and disease-specific DSSs would vary between rare diseases, and we found that this was the case. Endpoints in half the clinical studies for five of the ten rare diseases with one disease-specific DSS had an acceptable level of match to the surrogate disease phenotype. Endpoints in most of the clinical studies for the other five had an unacceptable level. We also expected that for rare diseases with more than one disease-specific DSS, the match between the fixed sample of clinical studies would be a worse match to the composite than the first disease-specific DSS, as we expected the latter to be a better representation of the disease phenotype. The number of indicators was greater in the composite than the first disease-specific DSS for all rare diseases with more than one disease-specific DSS, suggesting the expected increase in the body of knowledge. However, one subgroup of diseases was a better match and one subgroup was a worse match to the composite than the first disease-specific DSS. This suggested that the match between clinical study endpoints and the disease phenotype changed in different ways with respect to the body of knowledge.

### Rare diseases with one disease-specific disease severity score

Of rare diseases with one disease-specific DSS, achalasia (36 additional), systemic sclerosis (28) and systemic lupus erythematosus (24) contained the most indicators that were not present in their disease-specific DSSs. Variability of included indicators across studies within the same disease/condition was reflected in considerable rates of false positives and false negatives (Fig. 2). Misclassification appeared to favour higher false-positive than false-negative rates, driven by the mismatch between clinical study indicators and their respective disease-specific DSSs. Additionally, the rate of false positives decreased with increasing prevalence of severe disease, while false-negative rates increased. A high false-positive rate is concerning, because it suggests that affected clinical studies may be insensitive to changes in disease state either due to progression or intervention. Overall, false positives and negatives are equally damaging for clinical studies and match to the disease phenotype should be considered during study design.

Half the rare diseases with one disease-specific DSS (5/10) had at least one study that was a perfect match to the surrogate disease phenotype, demonstrating that the inclusion of a disease-specific DSS in a clinical study is not beyond reach. Development and utilisation of DSS in clinical studies would enable more consistent stratification, inclusion/exclusion of patients and efficacy evaluation. Consideration should also be given to the inclusion of more speculative or ‘redundant’ constructs alongside disease-specific DSSs to enable direct comparison, as was the case when comparing generic and disease-specific HRQoL tools [15]. This could provide useful information on the possible refinement of disease-specific DSSs.

### Rare diseases with more than one disease-specific disease severity score

In the first subgroup of rare diseases with more than one disease-specific DSS, the mean neutrality of clinical studies was higher when measured against the composite than the first disease-specific DSS, and this appeared to be driven by an increase in sensitivity. That is, as the number of indicators in the surrogate for the disease phenotype increased over time as a function of growth in the body of knowledge, clinical studies included a greater proportion of its indicators. This was not merely a function of the higher number of indicators increasing the probability of a match between the composite and clinical studies, because we did not find this pattern across all diseases. Rather, this suggested a convergence of knowledge whereby disease-specific DSS indicators generated through scientific research over time also showed up in the group of clinical studies assessed. We assumed that the sample of clinical studies would be static in terms of growth in the body of knowledge; however, it contained research spanning many years, so the body of knowledge that informed the composite may also have informed a proportion of the clinical studies in the sample. In Peirce’s convergence of truth and the mathematics upon which it is based, a convergence of knowledge on a construct observed alongside an increase in sample size can be taken as a sign of the validity of the knowledge generated [20, 21].

The convergence of knowledge between disease-specific DSSs and clinical studies in the first subgroup suggested that the disease phenotype operationalised by the indicators shared between them tended towards a more accurate representation of the disease phenotype over time, producing more accurate measures of disease severity. In the second subgroup of diseases, endpoint were a worse match to the composite, which we expected under the incorrect assumption that clinical studies would be unaffected by the increasing body of knowledge. Given our findings in the first subgroup, this can be better interpreted as a divergence of knowledge. If methods used in clinical studies and to develop disease-specific DSSs were sound, then this divergence may be fertile ground for hypothesis building and further knowledge generation [22]. Further research may examine qualitative differences between indicators in each disease, as our findings suggested that disease-specific DSSs in the first subgroup were more likely to contain indicators that were specific, measurable and objective and that were pathophysiological as well as behavioural and psychological. As we measured clinical studies as a homogenous group and did not separate them out into two timepoints, our findings cannot suggest that the changes in neutrality found represented a specific relationship between neutrality and time within the diseases studied. However, our methods were sufficient to demonstrate that changes in how the disease phenotype is operationalised over time affect the accuracy of clinical trial disease measurement and that this must be accounted for during the selection of endpoints.

The mean sensitivity of clinical studies for encephalitis remained constant at zero, suggesting that clinical studies included no indicators relevant to disease severity as defined by the disease-specific DSS at either time point, suggesting a divergence of knowledge regarding the operationalisation of disease phenotype between clinical studies and disease-specific DSSs. The Clinical Assessment Scale for Autoimmune Encephalitis is the only disease-specific DSS developed and validated for use in patients with encephalitis [23, 24]. Other DSSs for encephalitis included here were designed and validated to measure status epilepticus, a single intracranial complication that only covers part of the disease phenotype [25–27]. This could account for the lack of overlap of indicators between clinical studies and disease-specific DSSs at both timepoints. Additionally, heterogeneity of disease phenotype between and within rare disease subtypes, as is found to a great degree in encephalitis [28–30], may affect the accuracy and standardisation of disease-severity measurement in clinical trials [30].

### False positive and false negative rates

Substantial variation in false positive rate from min to max was found for each disease. This was expected and demonstrates that the probability of a false positive is influenced by endpoint selection and match to the Neutral list. Additionally, the false negative rate increased as disease prevalence increased. False positive and false negative rates are characterised by the positive and negative predictive values, respectively. Dependence of the positive and negative predictive values on the disease prevalence is well recognised in epidemiological literature, and NPV decreases with increasing prevalence. This is because the probability of a false negative increases with increasing prevalence due to the decrease in the number of true negatives in relation to the number of false negatives. That is, we expect a lower false negative rate at a lower disease prevalence, because a false negative suggests the presence of disease, which is low at a low prevalence. As prevalence increases, the probability of finding a true negative decreases.

### Implications

The inaccurate measurement of disease severity in clinical trials may result in patient misclassification [11–16, 30]. We measured the impact of neutrality via its components, sensitivity and specificity, on the probability of detecting false negative and false positive results at different disease prevalence rates. In a clinical trial setting (20% prevalence rate), in many diseases, the probability of a false positive was equal to one (the classification of a patient as ‘severe’ when they are ‘not severe’). If these disease-severity measures were used as inclusion criteria for trials, our findings suggested a high probability of including patients outside of the target population. Additionally, in many diseases, specificity was equal to zero, meaning that all indicators observed in clinical studies were irrelevant to disease severity. The detection of a treatment effect in these cases could result in the licensing of a medicine with little clinical significance to patients. If no treatment effect was detected, then trials may be abandoned, and effective medicines may be rejected at the regulatory stage, meaning that potentially life-changing medications may fail to reach patients, which is a recurrent problem in rare disease clinical trials and may be attributed to lack of neutrality in endpoint selection [31, 32]. Further, for these diseases, outcomes of relevance to disease severity may be underrepresented in the body of research, so patients may not benefit from ongoing evidence generation regarding the problems they deal with in their day-to-day lives. We observed a similar pattern of data at all prevalence rates that became more pronounced as prevalence increased, which was in line with findings for rare diseases with one disease-specific DSS.

### Limitations

First, we assumed that the disease-specific DSS was a surrogate for the disease phenotype, as it was the most accurate representation of the disease phenotype available. However, the disease phenotype is an empirically unattainable theoretical concept. This is likely to have resulted in an over-estimation of the neutrality of clinical studies in this study than if the disease phenotype was used as a comparison rather than disease-specific DSSs. Additionally, the heterogeneity of many rare disease is well documented. For this reason, DSSs do not represent an ideal match to the Neutral list. We propose that the Neutral list for a disease would account for disease heterogeneity and that this can be operationalised in endpoint selection through the use of expert opinion. Second, we assumed that indicators were independent of each other; however, associations may exist between indicators to varying degrees. Additionally, we did not control for the effect of time of publication of clinical studies, which may be reasonably expected to affect the number of indicators they shared with disease-specific DSSs to some degree (clinical studies published before studies in the composite may be less likely to contain their indicators, although this is not guaranteed, as disease-specific DSSs are generated based on existing bodies of knowledge shared by those who conduct clinical studies). The variation in the year of publication of disease-specific DSSs between diseases was not suggestive of a confound in respect of the effects noted in this study, and most disease-specific DSSs were published between 5 and 10 years before the analysis.

Validity of these findings as a reflection of the body of knowledge on a disease is limited by the robustness of our systematic review and is specific to the time of study. Validity of the results as a demonstration of the premise of the paper (that endpoints used in clinical studies vary from a theoretical and operationalised ‘ideal’ and may influence the probability of misclassification) is independent of this. One limitation of Neutral theory is that the Neutral list is a theoretical construct and is not empirically attainable. As such, to operationalise the concept, a surrogate for the Neutral list is needed. The fact that the Neutral list is empirically unobtainable does not negate the need for clinical endpoints to aspire to neutrality. On the contrary, the theory of Neutrality demonstrates the gap between existing clinical endpoints and the ideal set of endpoints that includes all relevant indicators and excludes all irrelevant indicators. This study demonstrates the gap between existing clinical endpoints and DSSs, acknowledged to be imperfect surrogates for the Neutral lists for each disease.

We do not propose that DSSs be used routinely for clinical trials in rare diseases. DSSs are of variable validity, some are designed for clinical bedside use rather than trial settings, and alternative endpoints may be identified during the course of a trial that would not be captured by a severity scale. Rather, we propose that rather than selecting endpoints pragmatically to suit the needs of the trial, endpoints should be selected that best represent the disease phenotype. DSSs provide a standardised operationalisation of a disease phenotype, and composite DSSs may be an especially helpful foundation, as they represent years of scientific effort. However, we propose that expert opinion could be an appropriate means of determining the Neutral list of clinical trial endpoints for a specific rare disease, with expert panels composed of academics, clinicians, researchers and patients. Selection of clinical trial endpoints in this way would provide greater standardisation, facilitating stakeholder decision-making and ultimately, we hope, a movement in drug development towards greater patient centricity and benefit.

## Conclusions

Our results confirmed the need to improve the accuracy of rare disease clinical study endpoints and suggested that the potential for accuracy in measuring disease severity increases as the body of knowledge on a disease increases. Clinical study endpoints in almost half the rare diseases in this study with more than one disease-specific DSS were a better match to the composite, while endpoints in the remaining rare diseases with more than one disease-specific DSS were a worse match. This suggested that sustained research efforts in some diseases resulted in the development of more accurate measures of disease severity. The application of Neutral theory could enhance the accuracy of endpoint selection in clinical trials and verify the accuracy and relevance of treatment effects as well as ensuring that the risk of misclassification during stratification, recruitment and the assessment of treatment effects is kept as low as possible. Further research may be beneficial to develop more accurate disease-severity measurements in Guillain-Barré syndrome, Behcet’s syndrome, Creutzfeldt-Jakob disease, atypical hemolytic uremic syndrome, Prader-Willi syndrome, Charcot Marie Tooth disease, Gaucher disease Type I, Huntington’s disease, Sjogren’s syndrome and Tourette syndrome.

## Supplementary Information

**Additional file 1: Table 1. **Generic and disease-specific search strings. **Fig 1.** Rates of false negatives and false positives at 20%, 50% and 80% prevalence of severe disease for the most and least Neutral studies (compared to first DSS) for each disease with more than one disease-severity scale. **Fig 2. **Rates of false negatives and false positives at 20%, 50% and 80% prevalence of severe disease for the most and least Neutral studies (compared to composite DSS) for each disease with more than one disease-severity scale. **Table 2. **The table presents the potential misclassification (median proportions of false positives and negatives along with 5th and 95th percentiles) for disease-specific severity score (DSS) for all diseases included in study results as compared to a choice of items. **Table 3. **The table presents the potential misclassification (median proportions of false positives and negatives along with 5th and 95th percentiles) for disease-specific severity score (DSS) for all diseases included in study results as compared to a choice of items. **Table 4. **The table presents the potential misclassification (median proportions of false positives and negatives along with 5th and 95th percentiles) for disease-specific severity score (DSS) for all diseases included in study results as compared to a choice of items. **Table 5. **Endpoints used in clinical studies for Fournier’s Gangrene showing distribution of endpoints within and outside Fournier’s Gangrene Severity Index. 

## Data Availability

All data generated or analysed during the current study are included in this published article and are available from the corresponding author on reasonable request.

## Abbreviation

DSS: Disease-severity score
