# Supplementary material for: Neutral theory: applicability and neutrality of clinical study endpoints where a disease-specific instrument is available
Source: BMC Med Res Methodol. 2023 May 20;23:121. doi: 10.1186/s12874-023-01947-z (PMC10199426; doi:10.1186/s12874-023-01947-z)
Supplement: Supplementary file 1 — Additional file 1: Table 1. Generic and disease-specific search strings. Fig 1. Rates of false negatives and false positives at 20%, 50% and 80% prevalence of severe disease for the most and least Neutral studies (compared to first DSS) for each disease with more than one disease-severity scale. Fig 2. Rates of false negatives and false positives at 20%, 50% and 80% prevalence of severe disease for the most and least Neutral studies (compared to composite DSS) for each disease with more than one disease-severity scale. Table 2. The table presents the potential misclassification (median proportions of false positives and negatives along with 5th and 95th percentiles) for disease-specific severity score (DSS) for all diseases included in study results as compared to a choice of items. Table 3. The table presents the potential misclassification (median proportions of false positives and negatives along with 5th and 95th percentiles) for disease-specific severity score (DSS) for all diseases included in study results as compared to a choice of items. Table 4. The table presents the potential misclassification (median proportions of false positives and negatives along with 5th and 95th percentiles) for disease-specific severity score (DSS) for all diseases included in study results as compared to a choice of items. Table 5. Endpoints used in clinical studies for Fournier’s Gangrene showing distribution of endpoints within and outside Fournier’s Gangrene Severity Index. [file 12874_2023_1947_MOESM1_ESM.docx]

**Supplemental information**

**Supplemental Table 1: Generic and disease-specific search strings**

|  | Search string |
| --- | --- |
| Generic | An automated search was conducted in Medline (PubMed) using the following generic search string to identify rare diseases with validated disease-specific DSSs: (rare OR uncommon OR infrequent OR orphan) AND (Illness* OR sickness* OR infection* OR malady* OR disorder* OR condition* OR infirmity* OR abnormality*) AND (severity AND (score OR scale OR index OR activity). |
| Specific | Variations of the following search string were used within Title/Abstract: [“disease name/term” #1” OR #disease name/term #2” *etc*] (*e.g.* “achalasia” OR “esophageal achalasia” OR “achalasia cardiae” OR “cardiospasm”) AND [“severity”] AND [“score” OR “index” OR “instrument” OR “tool” OR “scale”]. All search strings have been provided in the appendix. |

**Supplemental Figures 1 and 2**


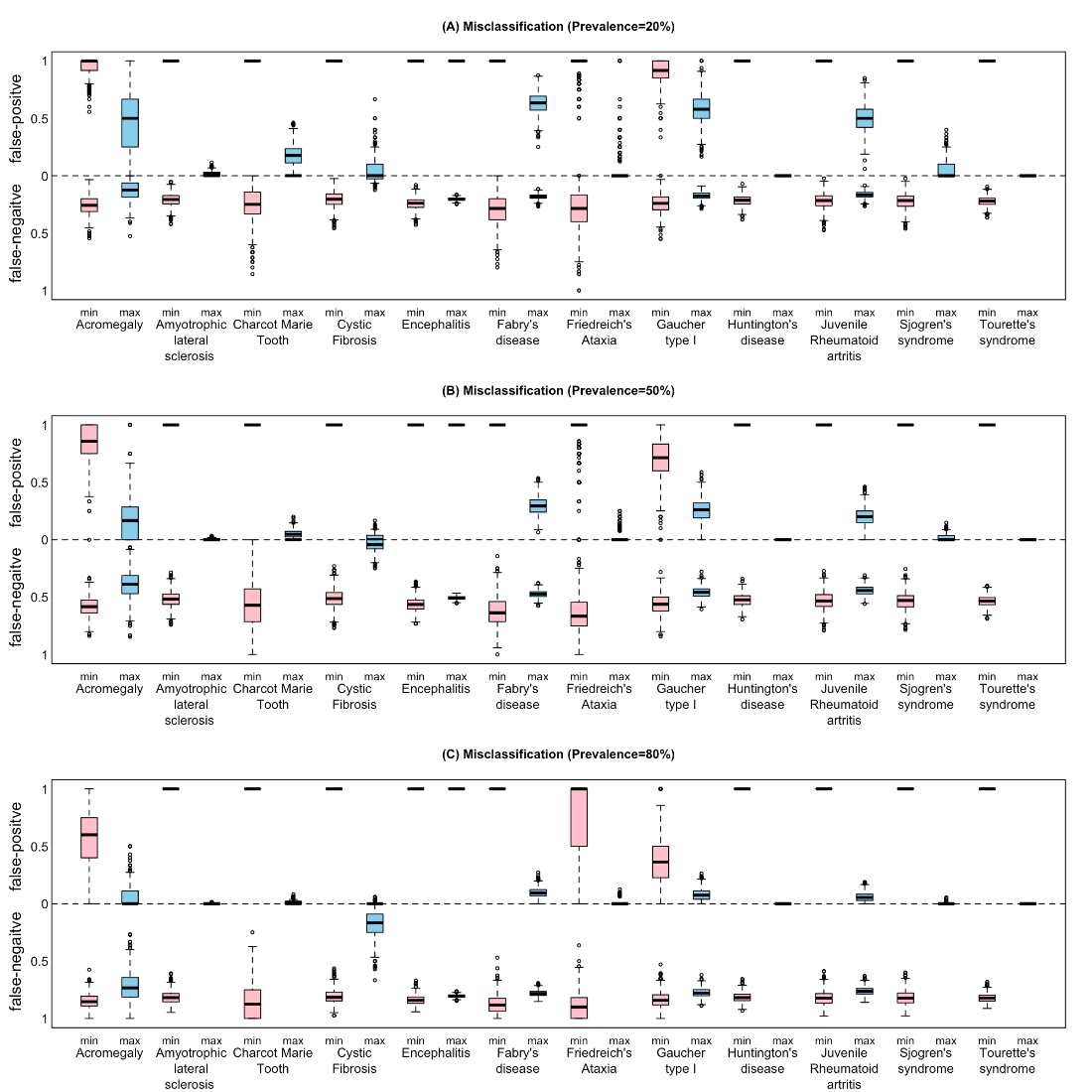


**Supplemental Fig 1. Rates of false negatives and false positives at 20%, 50% and 80% prevalence of severe disease for the most and least Neutral studies (compared to first DSS) for each disease with more than one disease-severity scale**


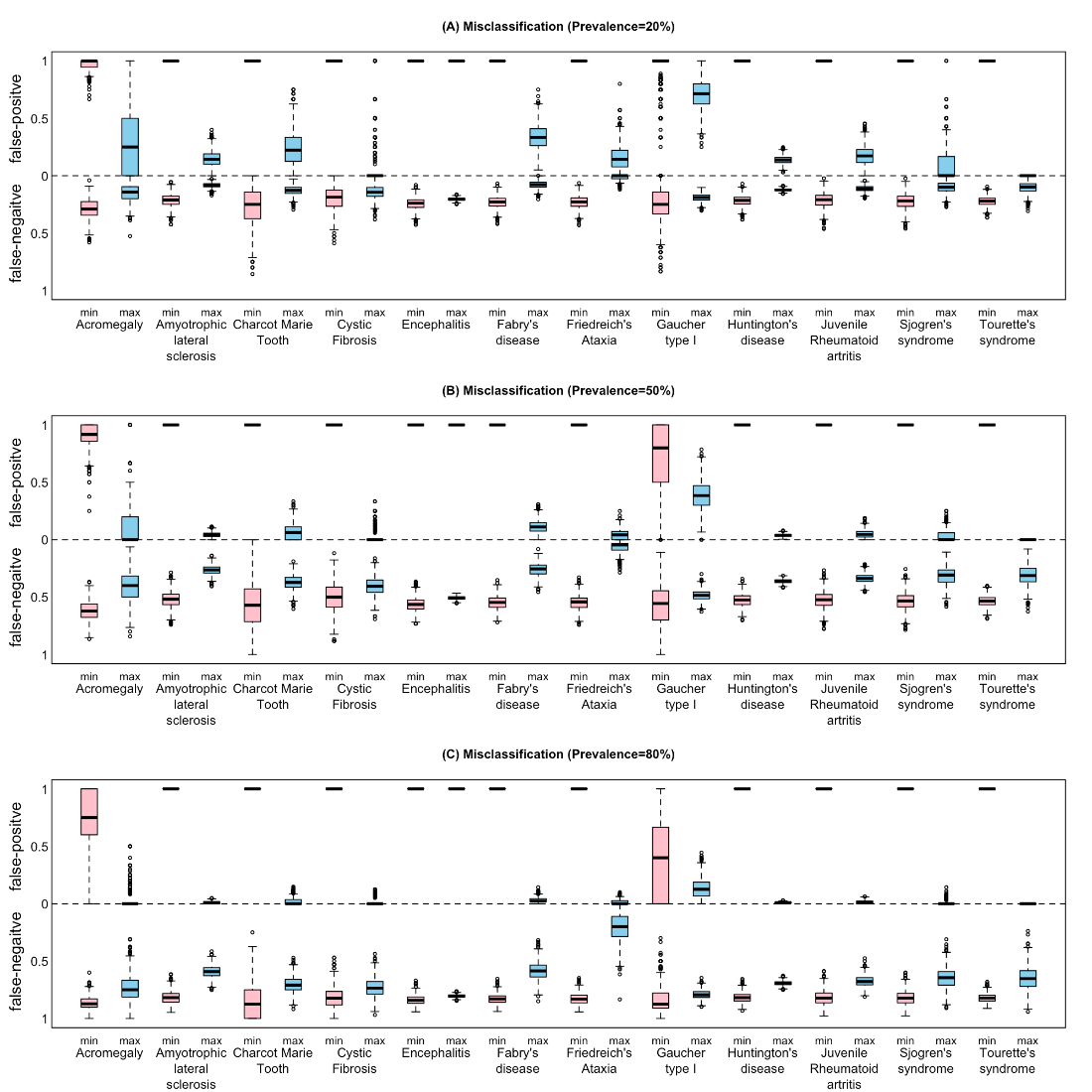


**Supplemental Fig 2. Rates of false negatives and false positives at 20%, 50% and 80% prevalence of severe disease for the most and least Neutral studies (compared to composite DSS) for each disease with more than one disease-severity scale**

**Supplemental Table 2: The table presents the potential misclassification (median proportions of false positives and negatives along with 5th and 95th percentiles) for disease-specific severity score (DSS) for all diseases included in study results as compared to a choice of items. The design of chosen items with respect to the DSS and the associated accuracy (neutrality, sensitivity, specificity) under neutral theory are also provided. In this main result table, the results are provided for each disease only for the two papers with lowest and highest accuracies. Prevalence of severe disease is 20%.**

| Disease name | Number of patients | Design | Accuracy | False negatives | False positives |
| --- | --- | --- | --- | --- | --- |
| Achalasia | 110 | (0, 5, 4, 31) | 0.86 (0.00, 0.86) | 0.22 (0.16, 0.29) | 1.00 (1.00, 1.00) |
| Achalasia | 24 | (4, 0, 0, 36) | 2.00 (1.00, 1.00) | 0.00 (0.00, 0.00) | 0.00 (0.00, 0.00) |
| Behcet’s disease | 21 | (0, 7, 20, 11) | 0.61 (0.00, 0.61) | 0.29 (0.09, 0.50) | 1.00 (1.00, 1.00) |
| Behcet’s disease | 50 | (2, 0, 18, 18) | 1.10 (0.10, 1.00) | 0.18 (0.09, 0.28) | 0.00 (0.00, 0.00) |
| Creutzfeldt disease | 29 | (0, 1, 25, 3) | 0.75 (0.00, 0.75) | 0.24 (0.11, 0.40) | 1.00 (1.00, 1.00) |
| Creutzfeldt disease | 54 | (3, 1, 22, 3) | 0.87 (0.12, 0.75) | 0.23 (0.12, 0.33) | 0.91 (0.71, 1.00) |
| Fournier’s gangrene | 100 | (0, 6, 9, 5) | 0.45 (0.00, 0.45) | 0.35 (0.25, 0.45) | 1.00 (1.00, 1.00) |
| Fournier’s gangrene | 50 | (9, 0, 0, 11) | 2.00 (1.00, 1.00) | 0.00 (0.00, 0.00) | 0.00 (0.00, 0.00) |
| Gullian-Barre Syndrome | 80 | (0, 6, 11, 3) | 0.33 (0.00, 0.33) | 0.43 (0.29, 0.56) | 1.00 (1.00, 1.00) |
| Gullian-Barre Syndrome | 8 | (10, 1, 1, 8) | 1.80 (0.91, 0.89) | 0.00 (0.00, 0.17) | 0.33 (0.00, 1.00) |
| Haemolytic disease | 93 | (0, 9, 12, 7) | 0.44 (0.00, 0.44) | 0.36 (0.24, 0.48) | 1.00 (1.00, 1.00) |
| Haemolytic disease | 41 | (1, 6, 11, 10) | 0.71 (0.08, 0.62) | 0.27 (0.14, 0.40) | 1.00 (0.83, 1.00) |
| Palmoplantar pustulosis | 188 | (0, 1, 3, 0) | 0.00 (0.00, 0.00) | 1.00 (1.00, 1.00) | 1.00 (1.00, 1.00) |
| Palmoplantar pustulosis | 24 | (3, 0, 0, 1) | 2.00 (1.00, 1.00) | 0.00 (0.00, 0.00) | 0.00 (0.00, 0.00) |
| Prader-Willi Syndrome | 85 | (1, 6, 27, 5) | 0.49 (0.04, 0.45) | 0.35 (0.23, 0.46) | 1.00 (0.94, 1.00) |
| Prader-Willi Syndrome | 77 | (3, 1, 25, 10) | 1.02 (0.11, 0.91) | 0.20 (0.12, 0.27) | 0.78 (0.50, 1.00) |
| Systemic Lupus Erythematosus | 834 | (1, 5, 23, 19) | 0.83 (0.04, 0.79) | 0.23 (0.21, 0.26) | 0.95 (0.92, 0.98) |
| Systemic Lupus Erythematosus | 11 | (24, 0, 0, 24) | 2.00 (1.00, 1.00) | 0.00 (0.00, 0.00) | 0.00 (0.00, 0.00) |
| Systemic Sclerosis | 84 | (0, 5, 10, 23) | 0.82 (0.00, 0.82) | 0.23 (0.15, 0.31) | 1.00 (1.00, 1.00) |
| Systemic Sclerosis | 15 | (10, 0, 0, 28) | 2.00 (1.00, 1.00) | 0.00 (0.00, 0.00) | 0.00 (0.00, 0.00) |

**Supplemental Table 3: The table presents the potential misclassification (median proportions of false positives and negatives along with 5th and 95th percentiles) for disease-specific severity score (DSS) for all diseases included in study results as compared to a choice of items. The design of chosen items with respect to the DSS and the associated accuracy (neutrality, sensitivity, specificity) under neutral theory are also provided. In this main result table, the results are provided for each disease only for the two papers with lowest and highest accuracies. In this main result table, the results are provided for each disease only for the two papers with lowest and highest accuracies. Prevalence of severe disease is 50%.**

| Disease name | Number of patients | Design | Accuracy | False negatives | False positives |
| --- | --- | --- | --- | --- | --- |
| Achalasia | 110 | (0, 5, 4, 31) | 0.86 (0.00, 0.86) | 0.54 (0.46, 0.62) | 1.00 (1.00, 1.00) |
| Achalasia | 24 | (4, 0, 0, 36) | 2.00 (1.00, 1.00) | 0.00 (0.00, 0.00) | 0.00 (0.00, 0.00) |
| Behcet’s disease | 21 | (0, 7, 20, 11) | 0.61 (0.00, 0.61) | 0.62 (0.42, 0.81) | 1.00 (1.00, 1.00) |
| Behcet’s disease | 50 | (2, 0, 18, 18) | 1.10 (0.10, 1.00) | 0.48 (0.35, 0.59) | 0.00 (0.00, 0.00) |
| Creutzfeldt disease | 29 | (0, 1, 25, 3) | 0.75 (0.00, 0.75) | 0.57 (0.41, 0.73) | 1.00 (1.00, 1.00) |
| Creutzfeldt disease | 54 | (3, 1, 22, 3) | 0.87 (0.12, 0.75) | 0.54 (0.42, 0.65) | 0.67 (0.40, 0.92) |
| Fournier’s gangrene | 100 | (0, 6, 9, 5) | 0.45 (0.00, 0.45) | 0.69 (0.59, 0.78) | 1.00 (1.00, 1.00) |
| Fournier’s gangrene | 50 | (9, 0, 0, 11) | 2.00 (1.00, 1.00) | 0.00 (0.00, 0.00) | 0.00 (0.00, 0.00) |
| Gullian-Barre Syndrome | 80 | (0, 6, 11, 3) | 0.33 (0.00, 0.33) | 0.75 (0.65, 0.85) | 1.00 (1.00, 1.00) |
| Gullian-Barre Syndrome | 8 | (10, 1, 1, 8) | 1.80 (0.91, 0.89) | 0.00 (0.00, 0.50) | 0.00 (0.00, 0.50) |
| Haemolytic disease | 93 | (0, 9, 12, 7) | 0.44 (0.00, 0.44) | 0.70 (0.60, 0.79) | 1.00 (1.00, 1.00) |
| Haemolytic disease | 41 | (1, 6, 11, 10) | 0.71 (0.08, 0.62) | 0.60 (0.45, 0.72) | 0.83 (0.57, 1.00) |
| Palmoplantar pustulosis | 188 | (0, 1, 3, 0) | 0.00 (0.00, 0.00) | 1.00 (1.00, 1.00) | 1.00 (1.00, 1.00) |
| Palmoplantar pustulosis | 24 | (3, 0, 0, 1) | 2.00 (1.00, 1.00) | 0.00 (0.00, 0.00) | 0.00 (0.00, 0.00) |
| Prader-Willi Syndrome | 85 | (1, 6, 27, 5) | 0.49 (0.04, 0.45) | 0.68 (0.58, 0.78) | 0.95 (0.85, 1.00) |
| Prader-Willi Syndrome | 77 | (3, 1, 25, 10) | 1.02 (0.11, 0.91) | 0.50 (0.40, 0.60) | 0.45 (0.14, 0.78) |
| Systemic Lupus Erythematosus | 834 | (1, 5, 23, 19) | 0.83 (0.04, 0.79) | 0.55 (0.52, 0.58) | 0.83 (0.77, 0.89) |
| Systemic Lupus Erythematosus | 11 | (24, 0, 0, 24) | 2.00 (1.00, 1.00) | 0.00 (0.00, 0.00) | 0.00 (0.00, 0.00) |
| Systemic Sclerosis | 84 | (0, 5, 10, 23) | 0.82 (0.00, 0.82) | 0.55 (0.46, 0.65) | 1.00 (1.00, 1.00) |
| Systemic Sclerosis | 15 | (10, 0, 0, 28) | 2.00 (1.00, 1.00) | 0.00 (0.00, 0.00) | 0.00 (0.00, 0.00) |

**Supplemental Table 4: The table presents the potential misclassification (median proportions of false positives and negatives along with 5th and 95th percentiles) for disease-specific severity score (DSS) for all diseases included in study results as compared to a choice of items. The design of chosen items with respect to the DSS and the associated accuracy (neutrality, sensitivity, specificity) under neutral theory are also provided. In this main result table, the results are provided for each disease only for the two papers with lowest and highest accuracies. In this main result table, the results are provided for each disease only for the two papers with lowest and highest accuracies. In this main result table, the results are provided for each disease only for the two papers with lowest and highest accuracies. Prevalence of severe disease is 80%.**

| Disease name | Number of patients | Design | Accuracy | False negatives | False positives |
| --- | --- | --- | --- | --- | --- |
| Achalasia | 110 | (0, 5, 4, 31) | 0.86 (0.00, 0.86) | 0.82 (0.76, 0.89) | 1.00 (1.00, 1.00) |
| Achalasia | 24 | (4, 0, 0, 36) | 2.00 (1.00, 1.00) | 0.00 (0.00, 0.00) | 0.00 (0.00, 0.00) |
| Behcet’s disease | 21 | (0, 7, 20, 11) | 0.61 (0.00, 0.61) | 0.89 (0.74, 1.00) | 1.00 (1.00, 1.00) |
| Behcet’s disease | 50 | (2, 0, 18, 18) | 1.10 (0.10, 1.00) | 0.79 (0.68, 0.88) | 0.00 (0.00, 0.00) |
| Creutzfeldt disease | 29 | (0, 1, 25, 3) | 0.75 (0.00, 0.75) | 0.85 (0.72, 0.96) | 1.00 (1.00, 1.00) |
| Creutzfeldt disease | 54 | (3, 1, 22, 3) | 0.87 (0.12, 0.75) | 0.83 (0.72, 0.91) | 0.33 (0.00, 0.67) |
| Fournier’s gangrene | 100 | (0, 6, 9, 5) | 0.45 (0.00, 0.45) | 0.90 (0.84, 0.95) | 1.00 (1.00, 1.00) |
| Fournier’s gangrene | 50 | (9, 0, 0, 11) | 2.00 (1.00, 1.00) | 0.00 (0.00, 0.00) | 0.00 (0.00, 0.00) |
| Gullian-Barre Syndrome | 80 | (0, 6, 11, 3) | 0.33 (0.00, 0.33) | 0.93 (0.87, 0.97) | 1.00 (1.00, 1.00) |
| Gullian-Barre Syndrome | 8 | (10, 1, 1, 8) | 1.80 (0.91, 0.89) | 0.07 (0.00, 1.00) | 0.00 (0.00, 0.20) |
| Haemolytic disease | 93 | (0, 9, 12, 7) | 0.44 (0.00, 0.44) | 0.90 (0.85, 0.95) | 1.00 (1.00, 1.00) |
| Haemolytic disease | 41 | (1, 6, 11, 10) | 0.71 (0.08, 0.62) | 0.86 (0.75, 0.94) | 0.50 (0.14, 1.00) |
| Palmoplantar pustulosis | 188 | (0, 1, 3, 0) | 0.00 (0.00, 0.00) | 1.00 (1.00, 1.00) | 1.00 (1.00, 1.00) |
| Palmoplantar pustulosis | 24 | (3, 0, 0, 1) | 2.00 (1.00, 1.00) | 0.00 (0.00, 0.00) | 0.00 (0.00, 0.00) |
| Prader-Willi Syndrome | 85 | (1, 6, 27, 5) | 0.49 (0.04, 0.45) | 0.90 (0.84, 0.95) | 0.80 (0.58, 1.00) |
| Prader-Willi Syndrome | 77 | (3, 1, 25, 10) | 1.02 (0.11, 0.91) | 0.80 (0.71, 0.87) | 0.17 (0.00, 0.45) |
| Systemic Lupus Erythematosus | 834 | (1, 5, 23, 19) | 0.83 (0.04, 0.79) | 0.83 (0.81, 0.85) | 0.55 (0.44, 0.66) |
| Systemic Lupus Erythematosus | 11 | (24, 0, 0, 24) | 2.00 (1.00, 1.00) | 0.00 (0.00, 0.00) | 0.00 (0.00, 0.00) |
| Systemic Sclerosis | 84 | (0, 5, 10, 23) | 0.82 (0.00, 0.82) | 0.83 (0.76, 0.90) | 1.00 (1.00, 1.00) |
| Systemic Sclerosis | 15 | (10, 0, 0, 28) | 2.00 (1.00, 1.00) | 0.00 (0.00, 0.00) | 0.00 (0.00, 0.00) |

**Supplemental Table 5: Endpoints used in clinical studies for Fournier’s Gangrene showing distribution of endpoints within and outside Fournier’s Gangrene Severity Index.**

|  | Fournier’s Gangrene Severity Index (FGSI) indicators | | | | | | | | | Additional clinical endpoints | | | | | | | | | | |
| --- | --- | --- | --- | --- | --- | --- | --- | --- | --- | --- | --- | --- | --- | --- | --- | --- | --- | --- | --- | --- |
| Study ID | **Temperature** | **Heart rate** | **Respiratory rate** | **White blood cell count (WBC)** | **Haematocrit** | **Serum sodium** | **Serum potassium** | **Serum creatinine** | **Serum bicarbonate** | **Leucocytes** | **Thrombocytes** | **CRP** | **Urea** | **Rate of anorectal fistula formation** | **SF-36** | **Mortality** | **systolic blood pressure** | **Clinical bleeding** | **Sepsis-related organ failure assessment (SOFA) scores** | **Use of renal replacement therapy (RRT)** |
| 2 | X | X | X | X | X | X | X | X | X |  |  |  |  |  | X |  |  |  |  |  |
| 3 | X | X | X | X | X | X | X | X | X |  |  |  |  |  |  |  |  |  |  |  |
| 4 |  |  |  |  |  |  |  |  |  | X | X | X | X |  |  |  |  |  |  |  |
| 6 |  |  |  |  |  |  |  |  |  |  |  |  |  | X |  |  |  |  |  |  |
| 7 |  |  |  |  |  |  |  |  |  |  |  |  |  |  | X | X | X | X | X | X |
